# Lightweight acoustic hyperbolic paraboloid diaphragms with graphene through self-assembly nanoarchitectonics

**DOI:** 10.1080/14686996.2024.2421757

**Published:** 2024-11-19

**Authors:** Mo Lin, Maxim Trubianov, Kou Yang, Siyu Chen, Qian Wang, Jiqiang Wu, Xiaojian Liao, Andreas Greiner, Kostya S. Novoselov, Daria V. Andreeva

**Affiliations:** a Institute for Functional Intelligent Materials, Department of Materials Science and Engineering, National University of Singapore, Singapore, Singapore; b School of Chemical Engineering and Light Industry, Guangdong University of Technology, Guangzhou, China; c School of Materials Science and Engineering, Tianjin University, Tianjin, People’s Republic of China; d Macromolecular Chemistry and Bavarian Polymer Institute, University of Bayreuth, Bayreuth, Germany

**Keywords:** Graphene oxide, polyacrylonitrile fibres, nanoarchitectonics, hyperbolic paraboloid shape, acoustic diaphragm

## Abstract

The paper presents a study on the fabrication of a lightweight acoustic hyperbolic paraboloid (HyPar) diaphragm using self-assembly nanoarchitectonics. The diaphragm is composed of a polyacrylonitrile (PAN) network combined with graphene oxide (GO) nanolayers. Spray coating is employed as a fabrication method, providing a simple and cost-effective approach to create large-scale curved diaphragms. The results demonstrate that the PAN/GO diaphragm exhibits acoustic performance comparable to a commercially available banana pulp diaphragm while significantly reducing weight and thickness. Notably, the graphene-based diaphragm is 15 times thinner and 8 times lighter than the commercial banana pulp diaphragm. This thinner and lighter nature of the graphene-based diaphragm offers advantages in applications where weight and size constraints are critical, such as in portable audio devices or acoustic sensors.

## IMPACT STATEMENT

This paper introduces a novel fabrication of a lightweight graphene-based acoustic HyPar diaphragm using self-assembly nanoarchitectonics, achieving significantly reduced thickness and weight while maintaining high acoustic performance.

## GRAPHICAL ABSTRACT

**Figure:**
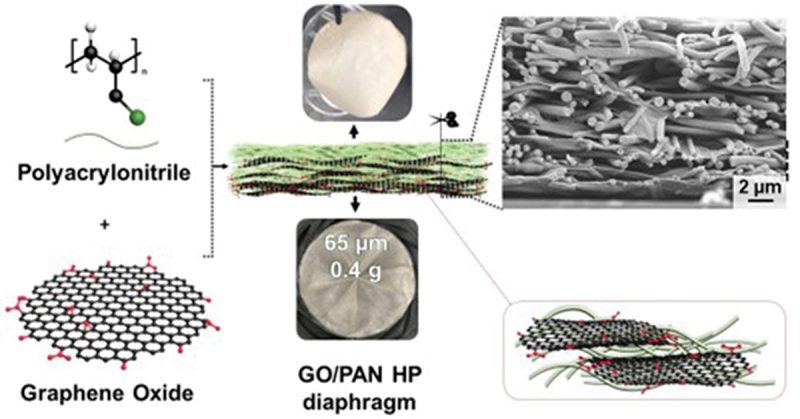

## 1. Introduction

Nanoarchitectonic materials represent a transformative approach in nanotechnology and materials science, where structures are meticulously designed and constructed at the nanoscale to achieve specific functions and properties [1]. By incorporating nanoscale building blocks such as low-dimensional carbon materials into bulk phases like polymers [2] or ceramics [3] and precisely designing their arrangement, it is possible to create composite materials that are both strong [4] and lightweight [5]. For instance, the incorporation of carbon nanotubes [6] or graphene [7] into polymer matrices significantly enhances their mechanical strength and stiffness [8] without adding significant weight. These nanoscale reinforcements form a strong network within the matrix, effectively distributing and transferring stress throughout the material [9]. This allows for the creation of lightweight materials with improved mechanical performance for aerospace [10], automotive [11], construction [12], and other industries where weight reduction is a critical factor.

In this study, we employed nanoarchitectonics to design a hyperbolic paraboloid (HyPar) diaphragm. A novel nanoarchitectonic material is assembled using graphene oxide (GO) nanolayers and polyacrylonitrile (PAN) fibre support. Graphene and its oxidized form GO are used to assemble thin and lightweight materials [13]. Its atomic thickness allows for the creation of diaphragms that are significantly thinner and lighter than conventional diaphragm materials [14].

Despite its thinness, graphene and GO possess high mechanical strength [15]. They have high tensile strengths, making them capable of withstanding substantial deformation and stress during diaphragm operation [16]. Graphene-based diaphragms offer a wide frequency response, making them suitable for various audio applications, including speakers and headphones [17]. The inherent stiffness and low mass of graphene enable diaphragms to respond rapidly and accurately across a broad range of frequencies [18].

The commercialization of graphene-based diaphragms is still in progress, with challenges like large-scale production, cost-effectiveness, and integration into existing audio systems [19]. Graphene’s properties, however, present an exciting opportunity for advancing acoustic technologies. GO, in particular, faces technological hurdles [20,21]. It is sensitive to humidity and temperature, which can degrade or alter its properties over time. Integration with polymeric materials and fibers provide cross-linking to maintain materials’ uniformity and structural integrity [22]. Overcoming these limitations is crucial and requires research into cost-effective synthesis, stabilization techniques, and efficient fabrication processes for the successful application of GO in diaphragm technology.

GO/PAN composites were assembled into a HyPar shape, which is a commercial patented diaphragm shape [23]. Studies in acoustic engineering highlight the benefits of complex geometric shapes, like the HyPar, in enhancing sound quality by controlling wave propagation and reducing distortions. However, traditional methods of assembly of GO membranes including vacuum filtration and spin coating are difficult to apply to such a complex geometry.

In this work, spray coating is applied as a versatile technique used to deposit thin films on curved substrates [24]. To increase the efficiency of spray coating method for thick curved membranes, we applied a spray coating with larger droplets. When utilizing large droplets in spray coating, several factors and considerations come into play to ensure optimal coverage and desired coating properties. This method allows us to prepare scalable uniform membranes with such complex curvatures and shapes as HyPar diaphragm, while significantly reducing the preparation time compared to the spray coating method with small droplets. Additionally, the curved membrane prepared by this method applies less strain than membrane prepared by mechanical deformation method [23], resulting in a uniform microstructure.

By controlling the arrangement of nanolayered GO and PAN fibres and optimizing their interactions, we created diaphragms with enhanced properties, such as improved strength-to-weight ratios and tailored acoustic characteristics. These diaphragms were then installed into a commercial speaker. The frequency responses of our diaphragms were measured and compared with simulation results.

## 2. Material and methods

### 2.1. Material and devices

A 0.4 wt.% graphene oxide (GO) water dispersion was obtained from Graphenea (U.S.A.) Inc. GO dispersions at a concentration of 0.3 mg/mL were sonicated (40 kHz, 55 W/L) for either 0.5 h or 3 h before use, resulting in monolayer flakes with average sizes of 2.1 ± 0.7 μm.

The electrospinning solution was prepared by dissolving 1.00 g of PAN powder in a hybrid solvent of 4.70 g dimethylformamide (DMF) and 0.965 g acetone. The solution was then loaded into a syringe fitted with a metal needle (0.6 mm diameter) and dispensed at a feed rate of 0.8 mL/h, controlled by a syringe pump. A positive direct current voltage of 16 kV was applied to the syringe needle, resulting in the continuous deposition of electrospun nanofibers on a grounded metal disc collector covered with aluminum foil. The distance between the spinneret and the collector was set at 30 cm. The electrospinning process was conducted at approximately 45°C with 10–15% humidity.

After electrospinning, the nanofiber membrane was heated in a vacuum oven at 60°C for 24 h. The resulting nanofiber membranes (approximately 1.00 g) were then cut into small pieces in liquid nitrogen and mixed with 500 mL of dioxane. The cooled mixture was further processed with a mixer (Robot Coupe Blixer 4, Rudolf Lange GmbH & Co. KG) at 4000 rpm until short fibrils formed. These short nanofibers were then freeze-dried for 48 h to obtain the final nanofiber powder.

3D Printing UV sensitive Standard Clear Resin (polyurethane-polyacrylate-based, 405 nm) and isopropyl alcohol (IPA, ≥99.8%) were purchased from ANYCUBIC and Fisher Scientific, respectively. The speaker used was a FOSTEX NF-01A, for which an AC voltage converter (Focket, ST-200VA) was used to step down the voltage from 220 V to 110 V, as the speaker’s recommended power supply is around 100 V.

### 2.2. 3D printing parameters

In this work, both the 3D models of the HyPar-shaped diaphragm and the template were designed using AutoCAD (Autodesk, Inc). We utilized Photo Workshop to slice the 3D template model and then imported it into an ANYCUBIC Photon Mono X 3D printer for fabrication. The template was designed to match the shape of the original diaphragm installed in the speaker, with dimensions set to 10.5 cm, slightly larger than the speaker’s required size to simplify installation.

To ensure stability during the printing process, support structures were added to the template. These supports had a diameter of 1.20 mm, a contact depth of 0.40 mm, and a contact diameter of 0.80 mm. By setting the auto-support angle to 51°, the software automatically generated an appropriate number of supports based on these parameters.

During the printing process, each layer was printed with a thickness of 0.05 mm. The normal exposure time for each layer was set to 2 s, followed by a 1-s off time. After printing was completed, the template was washed with isopropyl alcohol (IPA) for 15 min and then cured for an additional 15 min.

### 2.3. Simulation

The simulation was conducted in Ansys (Ansys, Inc). Ansys is a finite element analysis (FEA) software for simulating and analyzing of acoustic performance. We imported the 3D model of the diaphragm into Ansys and did structural simulations, harmonic response simulation and acoustic environment simulation.

### 2.4. Characterization

The microscopic structure of the as-prepared diaphragms was observed using a scanning electron microscope (SEM, Zeiss Sigma 300) operated at an acceleration voltage of 5 kV. Prior to imaging, the samples were sputter-coated with a thin layer of gold to enhance electrical conductivity and prevent charging during observation.

X-ray difractograms were collected using a Bruker D8 Advance diffractometer equipped with Cu Kα radiation (λ = 1.5406 Å) operated at 40 kV and 40 mA. The scans were performed over a 2θ range of 10° to 80°, with a step size of 0.02° and a scanning speed of 1°/min. Peak deconvolution and positions were determined and analysed using Origin Pro software.

Raman spectra were measured using a WITec Alpha 300 R confocal Raman microscope equipped with a 532 nm excitation laser. The laser power at the sample was set to 1 mW to prevent thermal damage. A 50× objective lens (numerical aperture NA = 0.75) was used to focus the laser on the sample. Each spectrum was acquired by accumulating 3 scans with an integration time of 15 seconds per scan. Data analysis was performed using WITec Project software. Peak deconvolution and positions were determined and analysed using Origin Pro software. Baseline correction was performed via WITec Project software’s built-in polynomial fitting function. A 9^th^-order polynomial was fitted to the background, and this baseline was subtracted from the raw spectra to isolate the Raman signal. The corrected Raman spectra were subjected to peak fitting using Lorentzian functions for both the D and G bands in Origin Pro software. The fitting parameters were adjusted to achieve the best fit with a coefficient of determination (R^2^) greater than 0.999. The peak positions were determined with an accuracy of ±1 cm^− 1^, based on the instrumental resolution and calibration.

Fourier transform infrared spectra were recorded using a SHIMADZU IRTracer-100 spectrometer in attenuated total reflectance (ATR) mode with a diamond crystal. Spectra were collected over the range of 4000–400 cm^− 1^ with a spectral resolution of 4 cm^− 1^, averaging 32 scans per sample to improve the signal-to-noise ratio. Background spectra were obtained under identical conditions and subtracted from the sample spectra to eliminate atmospheric interference.

The acoustic performance was measured using a calibrated microphone (Brüel & Kjær Type 4189) inside a custom-built anechoic chamber. The chamber was constructed with six layers of acoustic isolation polyurethane (PUR) foam (purchased from RS Components Pte Ltd) to minimize echoes and external noise. Sound pressure levels were recorded over a frequency range of 20 hz to 20 kHz. Data acquisition and analysis were performed using ARTA software and Origin Pro software, and the microphone was calibrated prior to measurements using a standard sound level calibrator.

To evaluate the physical and mechanical properties of the GO/PAN composites, we measured the density of diaphragms and the storage modulus using rectangular pieces which were cut from the diaphragms, and their lengths, widths, and thicknesses were recorded.

The thickness of the diaphragms was measured directly using a Mitutoyo Digimatic Micrometer (Series 293) with a resolution of 0.0001 mm (0.1 µm) and an accuracy of ±0.001 mm (1 µm). To account for any variations in thickness, measurements were taken at multiple points across diaphragm, and the average value was used for further analysis.

The density of the diaphragms was calculated by measuring both the mass and the volume of each sample. The mass was determined using an analytical balance with a precision of 0.1 mg. The volume was calculated by multiplying the measured average thickness (from the micrometer measurements) by the known surface area of the diaphragm. The coefficient of variation (CV) was used to assess the uniformity and consistency of the diaphragm properties defined as the ratio of the standard deviation (σ) to the mean (μ) of the measured values.

The mechanical properties of the diaphragms were measured by dynamic mechanical analyzer (DMA, TA Instruments). The film tension clamp was used to measure storage modulus from 0 to 100 hz.

## 3. Result and discussion

### 3.1. Diaphragm fabrication using spray coating assisted self-assembly

**Figure 1.**
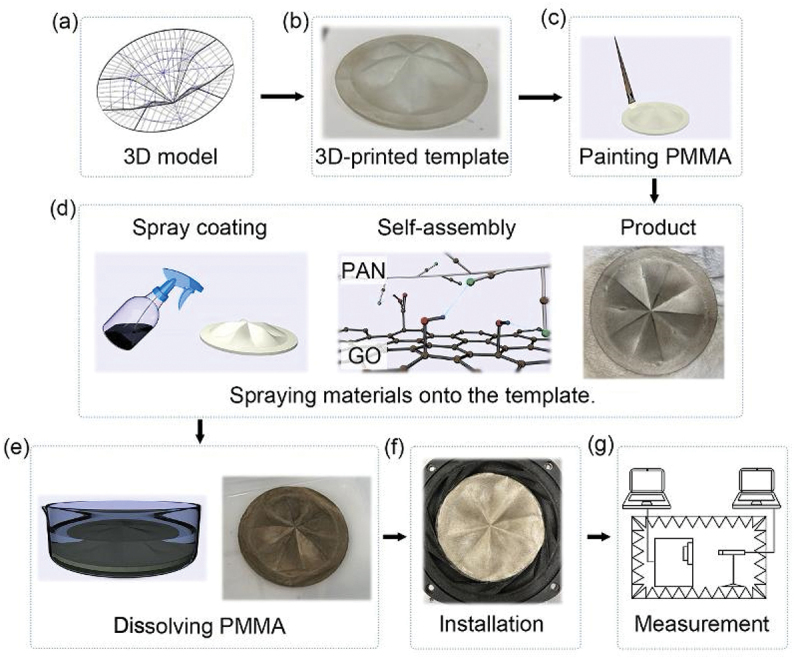
Steps to prepare a GO/PAN diaphragm installed speaker. a, building the 3D model of the template with CAD. b, preparing the template by 3D printing. c, painting PMMA onto the template. d, spraying materials onto the template. The hydrogen bonding stabilizes the assembly. e, submerging the membrane into acetone and obtaining free-standing GO-PAN membrane. f, installing the membrane into the speaker. g, conducting the measurement of the speaker in the soundproof chamber with a microphone.

The fabrication process, outlined in Figure 1, involved several key steps. Initially, a HyPar-shaped template was created using 3D printing technology to serve as the base for forming the diaphragms. Before the spray coating process, a sacrificial layer of polymethyl methacrylate (PMMA) was applied to the template’s surface using a paintbrush. The PMMA film was allowed to fully cure overnight, ensuring a smooth and uniform surface for the subsequent material deposition.

Next, a spray coating method was employed to apply a uniform dispersion of the GO/PAN composite material onto the surface of the template, ensuring controlled and consistent deposition. The spraying mixture was prepared by mixing the GO solution and PAN fiber suspension in a 1:1 ratio. The GO dispersion was first diluted in ethanol to a concentration of 0.2 mg/mL, while the PAN fibers were dispersed in ethanol at a concentration of 0.5 mg/mL. After the initial mixing, the dispersion was stirred for 3 h to ensure thorough and uniform mixing of the components. Once the mixture reached a homogeneous state, it was ready for application onto the 3D-printed template. A sprayer with a controlled droplet size of 0.44 ± 0.06 mm was used to apply the dispersion, achieving a consistent and precise coating on the template and ensuring uniformity in the final diaphragm.

Upon contact with the template, the sprayed droplets formed a thin wet layer on its surface, containing the dispersed material and serving as the foundation for the subsequent drying and solidification process. To expedite drying and facilitate solidification, a heat gun was used. After the liquid layer dried, additional droplets were sprayed onto the template. This process was repeated multiple times, resulting in films adhering to the PMMA layer on the template. By varying the number of spraying cycles, we were able to control the thickness of the diaphragms. After the spray coating process, the template was submerged in acetone to dissolve the sacrificial PMMA layer. The free-standing diaphragms were then collected from the acetone solution and dried in an oven at 80°C for 3 h before use.

### 3.2. The assembly of low-density GO/PAN composites

The average thicknesses and densities of the diaphragms are provided in Table 1. The thickness of each diaphragm was determined by the volume of the sprayed solution applied through repeated spraying and drying cycles. In our experiments, we prepared two diaphragms using 400 mL and 800 mL of the mixed solution, respectively. The average thickness of these diaphragms was measured to be 0.040 ± 0.004 mm (GO/PAN 40) and 0.065 ± 0.002 mm (GO/PAN 65).

**Table 1.** The physical properties of GO/PAN 40, GO/PAN 65 and GO diaphragm.

Sample	Thickness (mm)	Density (g/cm^3^)	Storage Modulus (GPa)	tan δ
GO/PAN 40	0.040 ± 0.004	0.56 ± 0.02	2.13 ± 0.48	0.070 ± 0.024
GO/PAN 65	0.065 ± 0.002	0.55 ± 0.04	2.25 ± 0.41	0.059 ± 0.016
GO 45	0.045 ± 0.002	1.26 ± 0.05	0.70 ± 0.17	0.076 ± 0.023

Notably, the average density of GO/PAN 65 was determined to be 0.55 ± 0.04 g/cm^3^, closely matching the density of GO/PAN 40 at 0.56 ± 0.02 g/cm^3^. This close consistency in density between the two samples indicates the stability and reproducibility of our fabrication method.

In addition to examining the average thicknesses and densities, we also evaluated the uniformity of these parameters across the diaphragms by calculating the coefficient of variation (CV). For GO/PAN 40, the CV for thickness was 0.05, indicating a relatively low level of variation. Similarly, GO/PAN 65 exhibited a CV of 0.04, suggesting an even higher degree of uniformity. Regarding density, GO/PAN 40 showed a CV of 0.03, while GO/PAN 65 had a slightly higher CV of 0.06. These low CV values for both thickness and density indicate that our spray coating method produces highly uniform diaphragms.

Furthermore, we compared the composite diaphragms with a GO diaphragm assembled using the same method. The density of the GO/PAN diaphragms (0.55 ± 0.04 g/cm^3^) were found to be lower than that of the pure GO diaphragm (1.26 ± 0.05 g/cm^3^) and the original banana pulp diaphragm (0.64 g/cm^3^) [23]. It is important to note that higher density can lead to reduced driver efficiency [18]. However, the lower density observed in our GO/PAN diaphragm makes it a promising candidate for acoustic applications, with the potential to enhance performance due to its favorable density profile.

### 3.3. Mechanical properties and enhancement of mass-to-stiffness ratio over commercial diaphragm

Oscillation tests were conducted at various frequencies to determine the storage modulus of the diaphragms. The measured storage modulus for the GO/PAN diaphragms was 2.13 ± 0.48 GPa and 2.25 ± 0.41 GPa, respectively. In contrast, a pure GO diaphragm fabricated using the spray coating method exhibited a storage modulus of only 0.70 ± 0.17 GPa. Furthermore, the electrospun-produced GO/PAN diaphragm exhibited a storage modulus of only around 0.1 GPa [25], highlighting the enhanced quality achievable with our fabrication method. This significant difference suggests that our GO/PAN diaphragm allows for faster propagation of sound waves, as it experiences less deformation under the same driving force [18].

While the storage modulus of the original diaphragm was reported as 11.02 GPa [23], which is higher than that of the GO/PAN diaphragm, the GO/PAN diaphragm still offers significant advantages due to its considerably reduced thickness and weight. The GO/PAN diaphragm has a higher mass-to-stiffness ratio of 4.21 × 10^−5^ kg m^2^ Pa^−1^ as compared to 3.36 × 10^−7^ kg m^2^ Pa^−1^ for the original. The mass-to-stiffness ratio is calculated as *m*/(*Et*^2^), where *m* is the mass in kg, *E* is the storage modulus in Pa, and *t* is the thickness in m. The mass-to-stiffness ratio of the GO/PAN diaphragm is 125 times higher than that of the commercial diaphragm. The reduced thickness and weight of the GO/PAN diaphragm allow for rapid and precise movements with less energy input, improving its response to acoustic signals, especially at higher frequencies.

We also measured the loss tangents (tan δ) of our samples, as shown in Table 1. Among the diaphragms, GO/PAN 65 exhibited the lowest tan δ value of 0.059 ± 0.016, indicating that it dissipates the least amount of energy in the form of heat. The tan δ of GO/PAN 40 (0.070 ± 0.024) is comparable to that of the pure GO diaphragm (0.076 ± 0.023), but as the diaphragm thickness increases, the tan δ decreases, further highlighting the benefits of a thicker diaphragm.

### 3.4. Self-assembly approach leads to the formation of a well-connected fiber network which provide structural support to the graphene oxide monolayers

**Figure 2.**
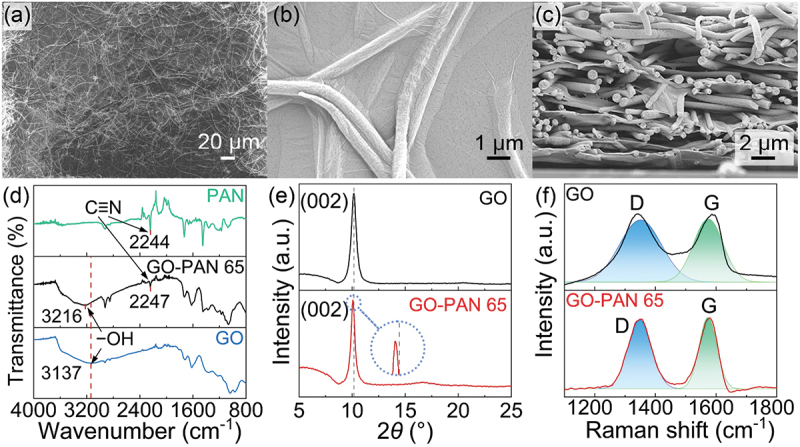
SEM, XRD and Raman characterization of GO/PAN diaphragm. a, the low magnification SEM image of GO/PAN diaphragm. b, the high magnification SEM image of GO/PAN diaphragm. c, the cross-section SEM images of GO/PAN diaphragm. d, the FTIR spectra of pure GO, PAN and GO/PAN diaphragm. e, the XRD diffractograms of pure GO and GO/PAN diaphragm. f, the Raman spectra of pure GO and GO/PAN diaphragm.

To elucidate the improved mechanical properties of our diaphragms, we conducted an evaluation of their morphology, structure, and composition. Scanning electron microscopy (SEM) images revealed that the fibers form a well-connected network, as shown in Figure 2a. The distribution of the fibers was found to be uniform throughout the diaphragm. Upon examination at higher magnification and through cross-section SEM imaging (Figure 2b, c), it was evident that the GO flakes form continuous nanolayers supported by the fibers. The presence of the fiber network explains the relatively lower density and higher storage modulus observed in our diaphragms compared to the GO diaphragms. The fibers act as a barrier, preventing the GO flakes from closely adhering to one another, resulting in a lower density in the GO/PAN film and remaining two-dimensionality of GO.

Additionally, the stiffness provided by the GO/PAN network contributes to the enhanced storage modulus of the diaphragms. This can be attributed to the cross-linking between the fibers and the GO flakes via hydrogen bonds. In the Fourier-transform infrared (FTIR) spectrum of the GO/PAN diaphragm (Figure 2d), the −OH absorption band (3216 cm^−1^) has a clear blue shift in comparison with that of GO (3137 cm^−1^). The absorption peak of the C≡N groups in the GO-PAN 65 (2247 cm^−1^) also shifts to higher wavenumbers comparing with the PAN fibers (2244 cm^−1^). Those shifts of the FTIR absorption bands indicates the existence of hydrogen bonds between the −OH groups of GO flakes and the C≡N groups of PAN fibers [26,27]. The presence of the cross-linking reinforces the overall structure and mechanical properties of the diaphragms.

In summary, the SEM analysis confirms that the diaphragms exhibit a network morphology composed of PAN fibers, which effectively support GO nanolayers. This morphology leads to a lower density and higher storage modulus in our GO/PAN diaphragms compared to pure GO diaphragms. The cross-linking via hydrogen bonding between the fibers and GO flakes revealed by FTIR measurements further contribute to the integrity of our composites and enhanced mechanical properties observed in our diaphragms.

The x-ray diffraction (XRD) was used to characterize the interlayer distance in GO nanolayers (Figure 2e). The diffractogram contains the peak of GO near 10° [28], indicating the interlayer distance of 8.74 ± 0.002 Å. The XRD peak in GO-PAN 65’s diffractogram slightly shifts to lower angle contrasted with that of the GO films, which proves that the existence of PAN fiber increased the distance between GO layers to 0.07 ± 0.002 Å. The measurement error Δ*d* in Å was calculated based on the precision of our XRD measurements. The instrumental angular resolution is 0.005° in 2θ, and the software provides an estimated error for peak positions of ± 0.002° in 2θ. Based on this and the Bragg’s Law the calculated error in d-spacing is approximately ± 0.002 Å.

In the Raman spectra (Figure 2f), the GO peaks around 1350 cm^−1^ and 1585 cm^−1^ [29] can be seen as well. The intensity ratio of D and G peaks (I_D_/I_G_) of GO-PAN 65 is 0.99 while the I_D_/I_G_ of pure GO is 1.01. A higher I_D_/I_G_ ratio suggests a higher degree of structural disorder, which can be attributed to the presence of functional groups, defects, or structural modifications [30]. Comparing the I_D_/I_G_ ratios of GO-PAN 65 and pure GO, we can observe that they are quite similar, indicating similar levels of disorder and suggesting that the composite diaphragms retain the structural characteristics of GO.

### 3.5. Replacing the commercial banana pulp diaphragm in FOSTEX speakers with a GO/PAN diaphragm

Following the preparation of the diaphragm, it was installed into the speaker. The central part and the edges of the diaphragm were securely glued to the support structure of the speaker. Any excess edges of the diaphragm were trimmed accordingly. To assess the performance, the original diaphragm made of banana pulp in a commercially available speaker (FOSTEX) was replaced with the fabricated GO/PAN diaphragms. This substitution allowed for a direct comparison of the acoustic characteristics between the original diaphragm and the newly developed diaphragms.

**Figure 3.**
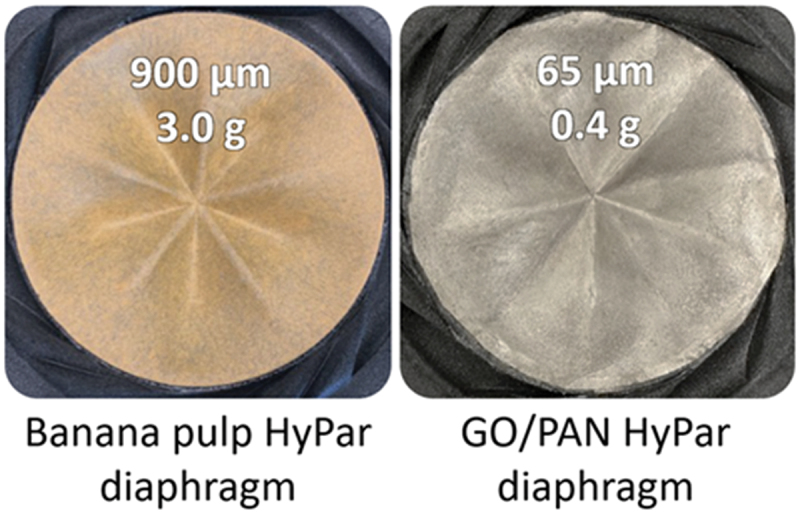
The digital photos of GO/PAN and commercial banana pulp diaphragms. Note, that the graphene-based diaphragm is significantly thinner and lighter, being 15 times thinner and 8 times lighter than the commercial banana pulp diaphragm.

Evaluation of physical properties shows that, the graphene-based diaphragm exhibits notable differences in thickness and weight compared to the commercial banana pulp diaphragm illustrated in Figure 3. Specifically, the graphene-based diaphragm is 15 times thinner and 8 times lighter than the banana pulp diaphragm. These findings highlight the potential advantages of using graphene-based materials in diaphragm applications.

**Figure 4.**
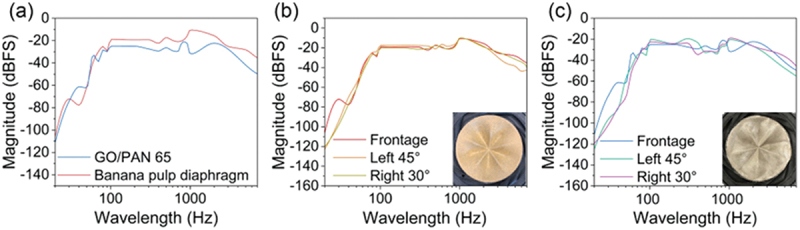
Frequency response of GO/PAN 65 and commercial banana pulp acoustic diaphragm. a, frequency response curve of GO/PAN 65 diaphragm and the original banana pulp diaphragm. Frequency response curve in different direction for the original banana pulp diaphragm (b) and GO/PAN 65 diaphragm (c).

The evaluation of the acoustic performance involved analyzing various parameters such as sound quality, frequency response, and overall efficiency. In Figure 4a, we can see that the GO/PAN 65 diaphragm gives similar uniformity as the original banana pulp diaphragm from 80 hz to 3000 hz. In Figure S1, GO/PAN 65, the thicker sample, showed better performance in comparison with GO/PAN 40. GO/PAN 65 diaphragm produced a more stable sound magnitude than GO/PAN 40 between 100 and 2000 hz. At the frequency above 2000 hz, the sound magnitude produced by GO/PAN 40 diaphragm decreased dramatically while the sound magnitude produced by GO/PAN 65 diaphragm dropped slowly. Overall, the GO/PAN 65 diaphragm showed more stable performance than GO/PAN 40 (Figure S2).

We measured the performance in different directions at certain distance (8 cm). For our GO/PAN 65 diaphragm (Figure 4c), the frequency responses detected from the sides of the speaker match well with the frequency response detected in front of the speaker. The magnitude of the sound only varies at the low frequency part, which won’t affect too much of our hearing experience. In comparison, the sound produced by the original banana pulp diaphragm (Figure 4b) has this fluctuation as well. In addition, the sound detected in front of GO/PAN 65 diaphragm is stronger than the sound detected in different directions. The increase of the thickness not only enhanced the sound’s frequency uniformity, but also raised the sound uniformity in different directions.

The performance of the GO/PAN diaphragms was compared with that of the original banana pulp diaphragm to evaluate the effectiveness of our fabrication approach and the potential improvements in acoustic performance. While the original diaphragm has a thickness of 0.84 mm, our diaphragm achieves similar performance with a significantly reduced thickness. This reduction not only results in material savings but also has the potential to lower production costs if applied to industrial manufacturing.

Moreover, the long-term stability and durability of graphene-embedded electrospun PAN nanofibers have been extensively studied, demonstrating robust and stable mechanical and thermal properties [31–34]. This ensures that our diaphragm is not only cost-effective but also reliable for prolonged use in practical applications.

### 3.6. Comparison between experimental acoustic performance and simulation results

**Figure 5.**
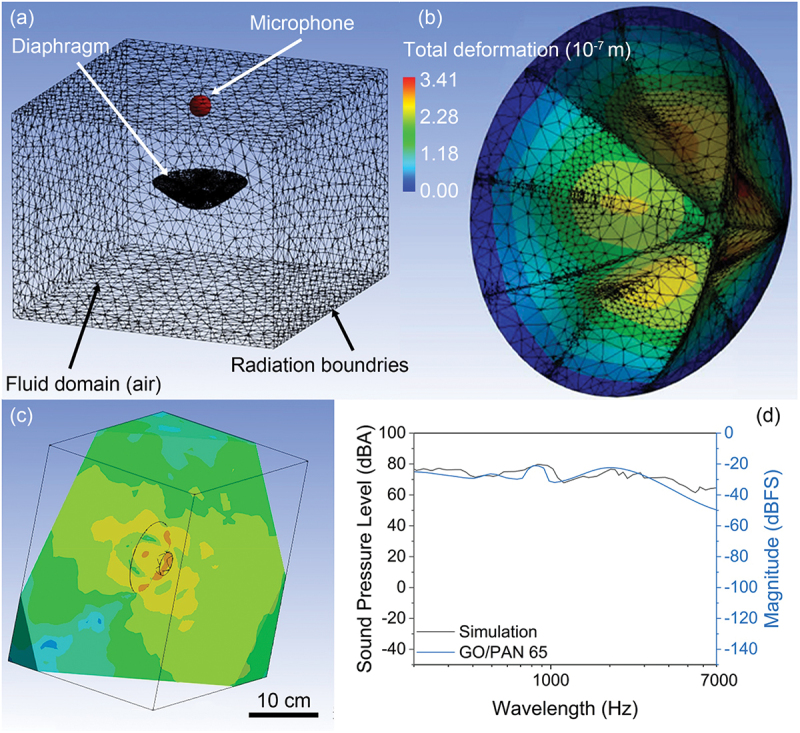
Simulation process and results. a, illustration of the simulation environment. b, frequency response of simulated diaphragm and the GO/PAN 65 diaphragm. c, frequency response in fluid domain. d, frequency response of simulated diaphragm and the GO/PAN 65 diaphragm.

In addition, we did simulations to predict the performance of the HyPar diaphragm in a fluid domain. The simulation was conducted in Ansys. Figure S3 shows the general process of the simulation. The models built in CAD were imported into this software for further computation. The geometry was defined, including volume domain, the material properties and fixed supports was set (Figure 5a). The modal analysis was carried out using the finite element method.

Firstly, we did structural simulations to obtain natural resonance frequencies and deformation modes of our HyPar diaphragm. Then, the harmonic response was carried out and the corresponding inherent frequencies of the weak positions of the model under the excitation of external cycle force were obtained (Figure 5b). Meanwhile, the deformation and velocity frequency response could be extracted either. After that, the diaphragm was simulated in the acoustic environment in the harmonic acoustic simulation part (Figure 5c). Here we imported the data extracted from the harmonic response and calculated the fluid domain under different frequencies. The enclosure was set to be air and the corresponding sound pressure level versus frequency could be obtained. During simulations, variables were material properties, diaphragm thickness, and diaphragm geometry.

The frequency response of the simulation and our GO/PAN 65 diaphragm is plotted in Figure 5d, we can see that between 200 hz and 2000 hz, our diaphragm has very similar performance trend with the simulated one, and that further proved the quality of our GO/PAN diaphragm.

## 4. Conclusion

In this research, we developed a scalable spray coating method to fabricate the GO/PAN acoustic diaphragm, providing a simple and cost-effective approach for producing large-scale curved diaphragms. Compared to the GO diaphragm made using the same method, the GO/PAN diaphragm exhibits lower density and higher storage modulus, both of which contribute to improved acoustic performance. By incorporating GO nanolayers into the PAN network, we achieved performance comparable to commercial diaphragms while significantly reducing weight and thickness. Additionally, previous studies have demonstrated the long-term stability and durability of graphene-embedded electrospun PAN nanofibers, highlighting their robust mechanical and thermal properties. This ensures that our diaphragm is not only cost-effective but also reliable for extended use in practical applications.

The GO/PAN diaphragm stands out for its reduced thickness and weight, being 15 times thinner and 8 times lighter than the commercial banana pulp diaphragm. This results in a mass-to-stiffness ratio of 4.21 × 10^− 5^ kg m^2^ Pa^− 1^, which is 125 times higher than the commercial diaphragm’s 3.36 × 10^− 7^ kg m^2^ Pa^− 1^. This balance is critical for diaphragm performance, enabling rapid and precise movements with minimal energy input. The lighter, thinner structure responds more efficiently to acoustic signals, especially at higher frequencies, requiring less force for the same acceleration. Overall, these findings suggest that the GO/PAN diaphragm has strong potential for sustainable next-generation acoustic devices.

## Supplementary Material

Supplemental Material
