# Supplementary material for: Lightweight acoustic hyperbolic paraboloid diaphragms with graphene through self-assembly nanoarchitectonics
Source: Sci Technol Adv Mater. 2024 Nov 19;25(1):2421757. doi: 10.1080/14686996.2024.2421757 (PMC11578420; doi:10.1080/14686996.2024.2421757)
Supplement: Supplemental Material [file TSTA_A_2421757_SM4153.docx]

Supplementary material

**Lightweight Acoustic Hyperbolic Paraboloid Diaphragms with Graphene through Self-Assembly Nanoarchitectonics**

*Figure S1*

Figure S1. Frequency response curve of GO/PAN 65 membrane and GO/PAN 40 membrane.

*Figure S2*

Figure S2. Frequency response curve in different direction for GO/PAN 40 membrane.

*Figure S3*


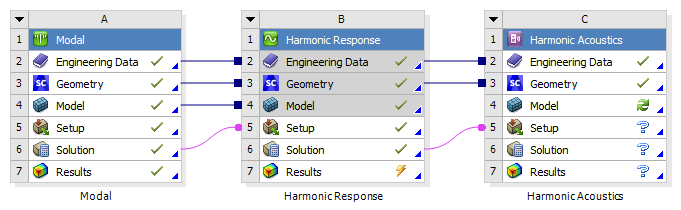


Figure S3. The general processes of the simulation.
